# Genome-Wide Identification, Characterization, and Expression Profiling Analysis of SPL Gene Family during the Inflorescence Development in *Trifolium repens*

**DOI:** 10.3390/genes13050900

**Published:** 2022-05-18

**Authors:** Jieyu Ma, Gang Nie, Zhongfu Yang, Sainan Ma, Jinwan Fan, Ruchang Hu, Feifei Wu, Xinquan Zhang

**Affiliations:** College of Grassland Science and Technology, Sichuan Agricultural University, Chengdu 611130, China; majieyu0224@163.com (J.M.); nieg17@sicau.edu.cn (G.N.); yangzf211@163.com (Z.Y.); masainan1997@163.com (S.M.); 15513999795@163.com (J.F.); huuruchang@163.com (R.H.); wufeifei_wu@163.com (F.W.)

**Keywords:** *Trifolium repens*, SPL gene family, phylogenetics, flowering regulation, expression patterns

## Abstract

*Trifolium repens* is the most widely cultivated perennial legume forage in temperate region around the world. It has rich nutritional value and good palatability, seasonal complementarity with grasses, and can improve the feed intake and digestibility of livestock. However, flowering time and inflorescence development directly affects the quality and yield of *T. repens*, as well as seed production. The *Squa promoter binding protein-like* (*SPL)* gene family is a plant specific transcription factor family, which has been proved to play a critical role in regulating plant formation time and development of flowers. In this study, a total of 37 *TrSPL* genes were identified from the whole genome of *T. repens* and were divided into nine clades based on phylogenetic tree. Seventeen *TrSPL* genes have potential target sites for miR156. The conserved motif of squamosa promoter binding protein (SBP) contains two zinc finger structures and one NLS structure. Gene structure analysis showed that all *TrSPL* genes contained SBP domain, while ankyrin repeat region was just distributed in part of genes. 37 *TrSPL* genes were relatively dispersedly distributed on 16 chromosomes, and 5 pairs of segmental repeat genes were found, which indicated that segmental duplication was the main way of gene expansion. Furthermore, the gene expression profiling showed that *TrSPL11, TrSPL13, TrSPL22*, and *TrSPL26* were highly expressed only in the early stage of inflorescence development, while *TrSPL1* and *TrSPL6* are highly expressed only in the mature inflorescence. Significantly, the expression of *TrSPL4* and *TrSPL12* increased gradually with the development of inflorescences. The results of this study will provide valuable clues for candidate gene selection and elucidating the molecular mechanism of *T. repens* flowering regulation.

## 1. Introduction

*T. repens* is the most important perennial legume forage in temperate regions originate from Europe [1]. As an allotetraploid plant, its two diploid ancestral species are *Trifolium occidentale* and *Trifolium pallescens* [2,3]. *T. repens* is mainly cultivated in perennial pasture together with other forage, and utilization through directly grazed or mechanically harvested into hay and silage [4]. It was a kind of high quality forage with rich protein and mineral content, good palatability, and high nitrogen fixation ability, which was beneficial to improve grassland quality, complement seasonal growth patterns of commonly forages, and promote the intake and digestibility of livestock [5,6]. However, flowering always reduces the number of axillary buds growing into branch stolons and leaf production [7], which leads to sharply dropped of the nutritional value and digestibility [8]. Therefore, delaying the flowering time and prolonging the vegetative growth period of *T. repens* will greatly improve the forage quality and yield [9]. Moreover, *T. repens* flowering at the suitable time and well-developed inflorescence are not only an important guarantee for seed production, but also increase the opportunity of resowing in the pasture under adverse conditions and enhancing the persistence [10,11]. Indeed, it is important to study the flowering time regulation and inflorescence development for future *T. repens* genetic improvement and breeding program; nevertheless, the molecular mechanism remain unknow due to the absence of omics data and limited availability of genome resource. The newly published genome article provides a possibility platform for the further study of *T. repens* [12].

Squa promoter binding protein–like (SPL) proteins are a plant-specific transcription factor family and operate on the characteristic genes of flower meristem, and participate in the formation and later development of flowers. The most leading feature of SPL proteins is that SBP-box (squamosa promoter binding protein) encodes a conserved protein domain with 76 amino acids [13]. The conserved SBP domain contains two zinc finger structures and one NLS structure, which are the structural basis for sequence-specific gene binding [14,15]. Although the *SPL* gene family had been widely studied in lots of species and the function of this kind of genes was also confirmed, there is no systematic exploration in *T. repens*. In snapdragon (*Antirrhinum majus*), *SBP1* and *SBP2* acted on the promoter region of flower meristem characteristic gene *SQUAMOSA* to control early flower development [16]. A total of 16 *AtSPL* genes were identified in *Arabidopsis thaliana*, and *AtSPL3* directly activates *LEAF*, *FRUITFULL*, and *APETALA1* to control the formation time of flowers [17]. Moreover, overexpressing *AtSPL10* showed an early flowering phenotype, and the triple loss-of-function mutants with its homologous genes *AtSPL2* and *AtSPL11* showed flowering later than wild type [18]. In terms of inflorescence development, *AtSPL8* showed significant functions in controlling pollen sac development and it is necessary for the normal development of spore tissue [19]. The loss of *AtSPL9* and *AtSPL15* function can lead to the change of inflorescence structure [20]. In rice (*Oryza sativa*), 19 *OsSPL* genes were identified [21], with the function of promoting the grain development to increase yield at the optimal expression level, and preventing the reversal of rice inflorescence during flowering [22,23]. There were 56 *TaSPL* genes in wheat (*Triticum aestivum*), and most of which regulated the development of inflorescence and spike [24]. In addition, 17 putative *DgSPL* genes were identified in orchardgrass (*Dactylis glomerata*), a common companion species of *T. repens* mixed grassland, which were mainly involved in vegetative to reproductive growth transition, flower development, and flowering regulation [25]. In *Medicago truncatula*, a total of 23 *MtSPL* genes were identified which are involved in the development of seed pods, especially the formation of thorns on pods [26].

It is critical to explore the white clover flowering time regulation and inflorescence development mechanism for its genetic improvement and utilization. Genome-wide identification, characterization, and expression profiling analysis of *SPL* gene family in *T. repens* will be an important basic work for providing valuable clues of candidate gene selection and breeding program. In this study, the *TrSPL* gene family was identified and comprehensively analyzed, including SBP conserved domain comparation, conserved motif composition, gene structure annotation, and chromosome location distribution. The evolution of *TrSPL* genes were preliminarily predicted by studying the intraspecific replication events, phylogenetic analysis and collinearity analysis with other plant species. Furthermore, an expression profiling analysis of *TrSPL* genes at different flower development stages were constructed.

## 2. Materials and Methods

### 2.1. Genome-Wide Identification of TrSPL Genes

The *T. repens* genome resource information came from previous study [12] and all files were provided by Stig Uggerhøj Andersen from Aarhus University. The hidden markov model (HMM) file of SBP domain (pf03110) was download from Pfam database [27] (pfam.xfam.org/, accessed on 5 March 2021). The downloaded HMM file was taken as the query sequence to search the protein sequence data of *T. repens* by using HMMER3.0. The obtained proteins were aligned by ClustaW (E-value < 1 × 10^−20^) and the SBP HMM file was rebuilt by hmmbuild in HMMER 3.0. Finally, SBP HMM of *T. repens* was used to identify SPL protein in *T. repens* genome, and the cut-off value was set to 0.01 [28]. In order to ensure that all candidate genes contain SBP domain, the NCBI Conserved Domain Search website was used for further confirmation (Conserved Domains Database (CDD) and Resources (nih.gov), accessed on 5 May 2021). The isoelectric point and relative molecular weight data were obtained using the Expasy website (Compute pI/MW—SIB Swiss Institute of Bioinformatics | Expasy, accessed on 5 May 2021). The subcellular localization information of *TrSPL* genes were analyzed and predicted online by BUSCA tool (BUSCA—Bologna Biocomputing Group (unibo.it), accessed on 6 May 2021). The psRNATarget website was used to predicted of miR156-targeted *TrSPL* genes (http://plantgrn.noble.org/psRNATarget/home, accessed on 2 May 2022). Cis acting element analysis was predicted and visualized by using online tools PlantCARE and TBtools software (http://bioinformatics.psb.ugent.be/webtools/plantcare/html/, accessed on 7 May 2021) [29].

### 2.2. Phylogenetic Analyses and Classification of TrSPL Genes

Genes and protein sequences of *A. thaliana*, *M. truncatula*, *Trifolium pratense*, *and G. max SPL* gene family are downloaded from TAIR (https://www.arabidopsis.org/download/index-auto.jsp?dir=%2Fdownload_files%2FSequences%2FAraport11_blastsets, accessed on 10 June 2021), the *M. truncatula* Genome Database (http://www.medicagogenome.org/, accessed on 10 June 2021), Ensembl website (https://plants.ensembl.org/index.html, accessed on 10 June 2021), and the *G. max* Genome Database (https://www.soybase.org/, accessed on 15 June 2021), respectively. Multiple sequence alignment of SPL protein sequences was performed by Log-Expectation (MUSCLE) in MEGA v6.0 [30]. Eventually, the phylogenetic tree was constructed by the neighbor-joining method of MEGA v6.0 software, and the bootstrap replications value was 1000.0.

### 2.3. Gene Structure and Motif Analysis of TrSPL Genes

Jalview software for SBP conservative sequence alignment of *T. repens* (http://www.jalview.org/, accessed on 10 September 2021), two zinc finger structures and one NLS structure are marked in the figure. The conserved motifs of *T. repens* are analyzed by MEME Suite 5.14 (Introduction—MEME Suite (meme-suite.org), accessed on 11 September 2021) [31], and the details of each conserved motif are also derived from the same website. The maximum number of motifs are set as 10. The program Visualize MEME/MAST Motif Pattern of Tbtools software was employed for conservative motif visual analysis. Gene structure analysis used the gene sequence file of *TrSPL* genes to analyze and visualize in TBtools software Visualize Gene Structure function, showing the CDS sequence, SBP conserved domain, and ANK conserved domain and intron.

### 2.4. Chromosomal Locations and Synteny Analysis of TrSPL Genes

The chromosome location information of *TrSPL* genes were obtained from the genome annotation file. The Gene Location Visualize From GTF/GFF function of Tbtools software was used for gene chromosome mapping and visual analysis. The intraspecific collinearity circle map of *TrSPL* genes were analyzed by using the Dual Synergy Plot for McscanX function of TBtools and visualized by the Advanced Circos function. The genome data of *A. thaliana* were downloaded from TAIR (https://www.arabidopsis.org/download/index-auto.jsp?dir=%2Fdownload_files%2FSequences%2FAraport11_blastsets, accessed on 13 September 2021). The genome data of *M. truncatula* were downloaded from the *M. truncatula* Genome Database (http://www.medicagogenome.org/, accessed on 10 October 2021). The genome data of *Trifolium pratense* were downloaded from Ensembl website (https://plants.ensembl.org/index.html, accessed on 15 October 2021). The genome data of *G. max* were downloaded from the *G. max* Genome Database (https://www.soybase.org/, accessed on 19 October 2021). Drawing with Dual Synteny Ploter function of TBtools.

### 2.5. Material Culture, Sampling and qRT-PCR

*T. repens* Seed Haifa is provided by Beijing MAMMOTH SEED company, with the registration number of 249. The same genotype material Haifa is used for asexual propagation by cutting in pot (the diameter is 16.5 cm, the height is 10 cm, and the bottom diameter is 12 cm). The growth environment of *T. repens* is a growth chamber with a Photoperiod of 14 h at 22 °C and a dark period of 10h at 20 °C. *T. repens* after flowering, fresh inflorescences in different developmental states are taken and stored in the freezing tube. (T1, Immature inflorescence; T2, Inflorescences in which no floret was open; T3, Inflorescences in which outermost circle of florets were open; T4, Inflorescences in which 50% of florets were open; and T5, Mature inflorescence). Three biological replicates per sample. The fresh sample was immediately put into liquid nitrogen and long-term preservation in a −80 °C refrigerator. Total RNA was extracted using the Hipure HP Plant RNA Mini Kit (Magen). The obtained RNA was reverse transcribed into cDNA using the MonScript^TM^ RTIII ALL-in-One Mix with dsDNase (Monad) kit. Primer design used primer5 software and Tr β- Actin as internal parameter [32]. All primers and internal paramenter information have been given in the attachment (Table S1). qRT-PCR was performed with Bio-Rad CFX96 instrument, and used SYBR^®^ green real-time PCR master Mix test kit. The qRT-PCR procedure as follow:30 s at 95 °C, denaturation (95 °C for 10s), anneal/extension (55 °C for 30 s), for 40 cycles, melting curve detection (65–95 °C). Each gene performed three biological and three technical repeats at each inflorescence development stage. 2^(^^−^^ΔΔCt)^ analysis method was used to calculate the relative expression of 16 *TrSPL* genes, and finally the expression maps of 16 *TrSPL* genes in five different flower development stages were obtained.

## 3. Results

### 3.1. Genome-Wide Identification of T. repens SPL Genes

**Figure 1:**
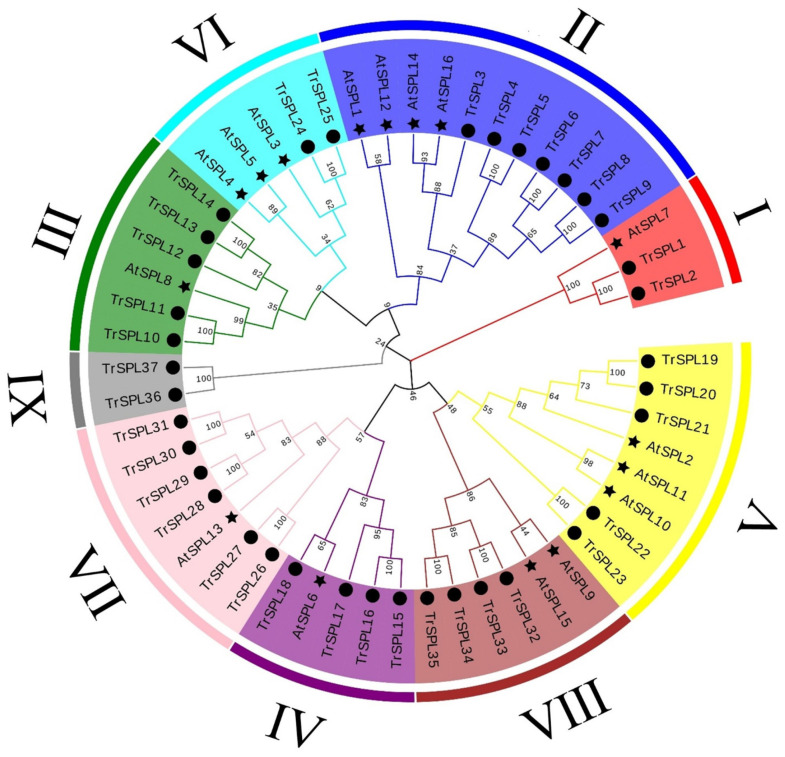
Phylogenetic analysis of SPL proteins in *A. thaliana* and *T. repens*. Phylogenetic trees were plotted using the neighbor-joining (NJ) method with a bootstrap value of 1000. Fifty-three genes were divided into nine clades (I–IX) and identified with different colors. The black circle represents *T. repens*, and the black pentacle represents *A. thaliana*.

Based on the *T. repens* genome resources, putative *TrSPL* genes were preliminarily identified by performing HMM (hidden Markov model) search (SBP domain, PF03110) from Pfam database. Subsequently, 38 *TrSPL* genes were identified after removing redundant sequences preliminarily. However, owing to the incomplete of SBP structure in gene *chr13.jg763.t1*, 37 genes with highly conserved SBP domain were retained (Table 1). According to the phylogenetic tree group numbering sequence of *T. repens* and *A. thaliana* (Figure 1), 37 *TrSPL* genes were named as *TrSPL1*–*TrSPL37*, respectively (Table S2). The isoelectric point (PI) of 40.5% of proteins was less than 7, with the lowest PI of *TrSPL5* was 5.72, and the highest PI of *TrSPL14* and *TrSPL33* was 9.41. The protein sequence length (aa) of all TrSPL proteins ranged from 1053 (*TrSPL6*) to 124 (*TrSPL12*) amino acids with an average of 474. The relative molecular weight (MW) ranged from 116204.16 Da to 14269.05 Da and the corresponding gene was consistent with the length of protein sequence. Subcellular localization results showed that 37 *SPL* genes in *T. repens* were located in the nucleus (30 genes), chloroplast (2 genes), plasma membrane (2 genes), and endomembrane system (3 genes), respectively. The basic data of *TrSPL* gene family varied widely, which indicated that diverse function of these genes.

### 3.2. Phylogenetic Analyses and Classification of the TrSPL Gene Family

**Figure 2:**
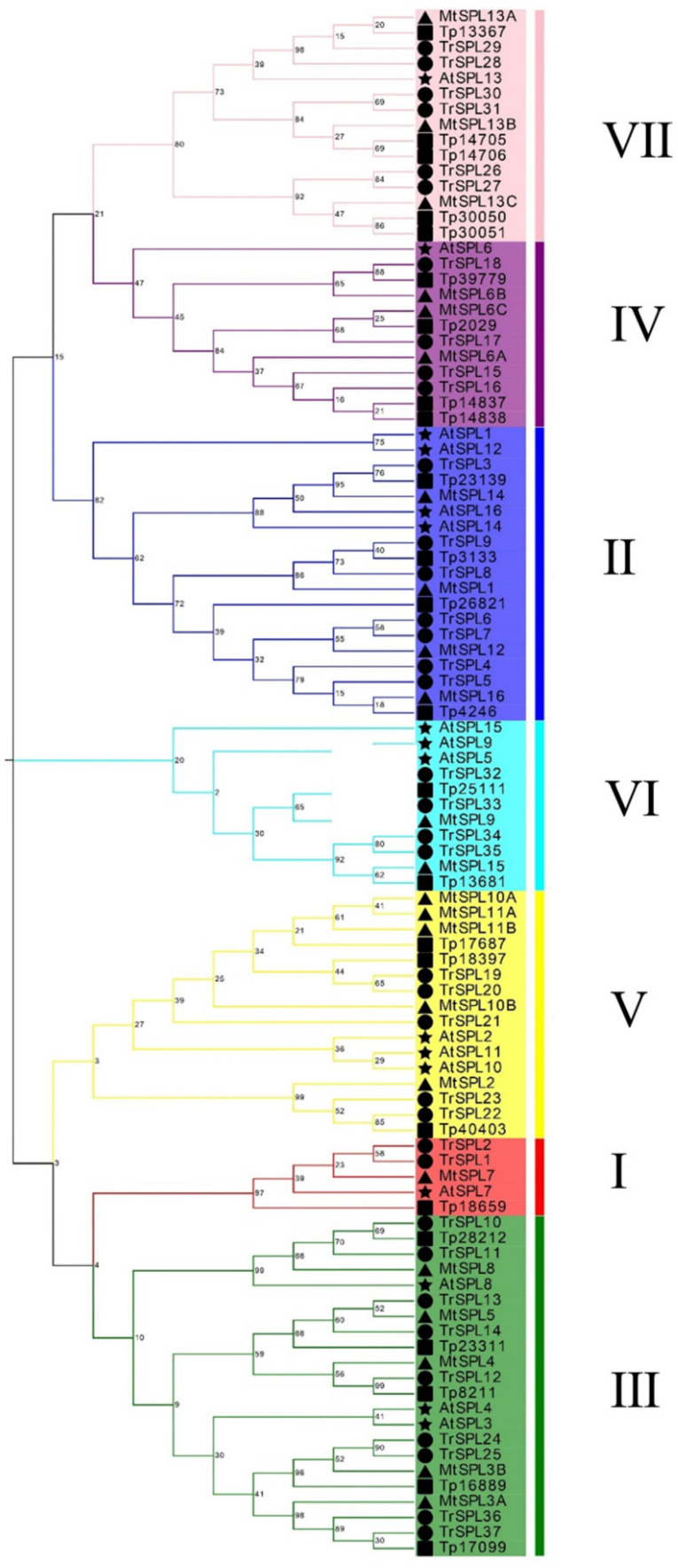
Phylogenetic analysis of SPL proteins in *A. thaliana*, *T. repens Trifolium pratense*, and *M. truncatula*. Phylogenetic trees were plotted using the neighbor-joining (NJ) method with a bootstrap value of 1000. A total of 100 genes were divided into 7 clades (I–VII) and identified with different colors. The black circle, pentacle, square and triangle represent *T. repens*, *A. thaliana*, *Trifolium pratense*, and *M. truncatula* respectively.

For the classification of the *TrSPL* gene family, a Neighbor-joining (NJ) tree (with 1,000 bootstraps) of 16 *A. thaliana SPL* genes and 37 identified *TrSPL* genes was constructed (Figure 1). The results showed that 53 genes were divided into 9 clades, and all 16 *SPL* genes in *A. thaliana* were distributed in 8 main *SPL* evolution clades named as I to VIII [33,34]. There were two *TrSPL* genes grouped into *T. repens*-specific clade named in IX, which indicated that potentially emerged after the divergence between two species. Moreover, in order to explore the evolutionary relationship of *SPL* gene families among the related species of *T. repens*, 24 *SPL* genes of red clover (*Trifolium pratense*) and 23 *SPL* genes of *M. truncatula* were analyzed together with white clover and *A. thaliana* (Figure 2). A total of 100 *SPL* genes were divided into 7 clades (I–VII), and each evolutionary clade contained all 4 species. The results showed that *SPL* gene distance between three legumes species were closer, while farther than *A. thaliana*. Multi species phylogenetic tree revealed that *SPL* gene family was relatively conservative in evolutionary direction.

SPL transcription factors are targeted for cleavage and/or translational repression by microRNA156s [35].The miR156/SPL module is involved in the regulation of flowering time, inflorescence development and yield improvement [17,36,37]. Among 37 *TrSPL* genes, 17 *TrSPL* genes contain miR156 complementary recognition sites, which may be regulated by miR156 targeting (Table S3).

Cis-acting elements are important in regulating gene expression. Analysis cis acting elements of 37 *TrSPL* genes (upstream 2000 bp) showed that *TrSPL* genes contained a large number of action elements in response to light (light response element, circadian rhythm regulation element, and phytochrome down-regulation response element) and hormones (abscisic acid response element, gibberellin response element, plant auxin response element, salicylic acid response element, and methyl jasmonate response element). In addition, there were some other elements in response to external stress, such as low temperature response element, defense and stress response element, and wounding response element. (Table S3; Figure S1).

### 3.3. Sequence Feature and Gene Structure of TrSPL Genes

**Figure 3:**
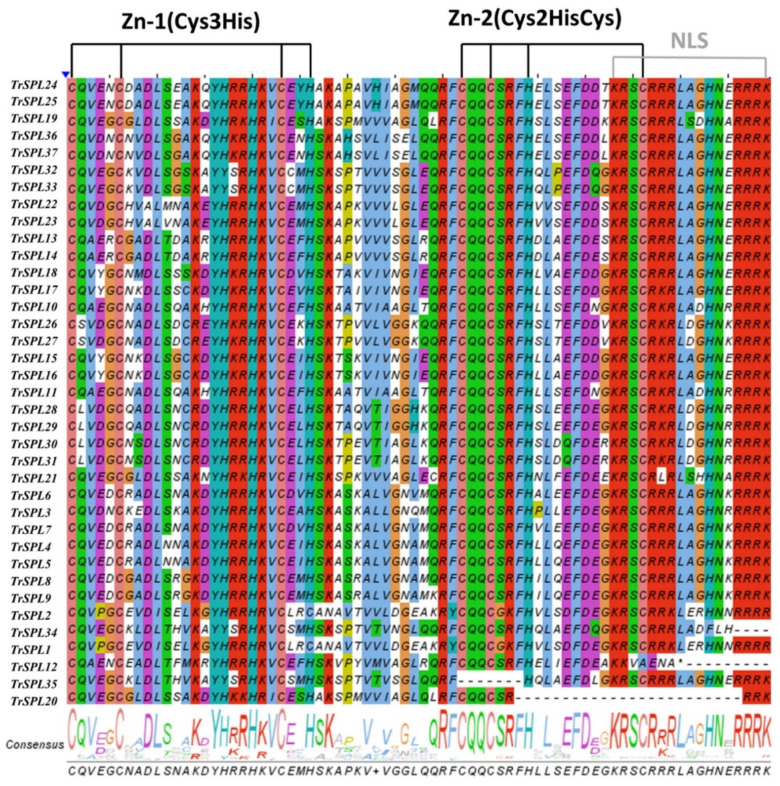
Sequence alignment and logo of SBP domain from *T. repens*. Two Zn-finger structures (Zn-1, Cys3 His; Zn-2, Cys2HisCys) and one NLS structure have been marked.

The full-length protein sequence of *T. repens SPL* genes were used for sequence alignment. The SBP domain was highly conserved in the *TrSPL* gene family (Figure 3). All SBP domains contained two zinc finger structures and a nuclear localization signal (NLS), along with absence of some small fragments from *TrSPL12*, *TrSPL 20*, *TrSPL 34*, and *TrSPL 35*. Besides, the motif of first *TrSPL* gene zinc finger for clade I (Cys-Cys-Cys-Cys) was different from that in the other clades (Cys-Cys-Cys-His), which was consistent with *M. truncatula* and other species [26,38,39].

**Figure 4:**
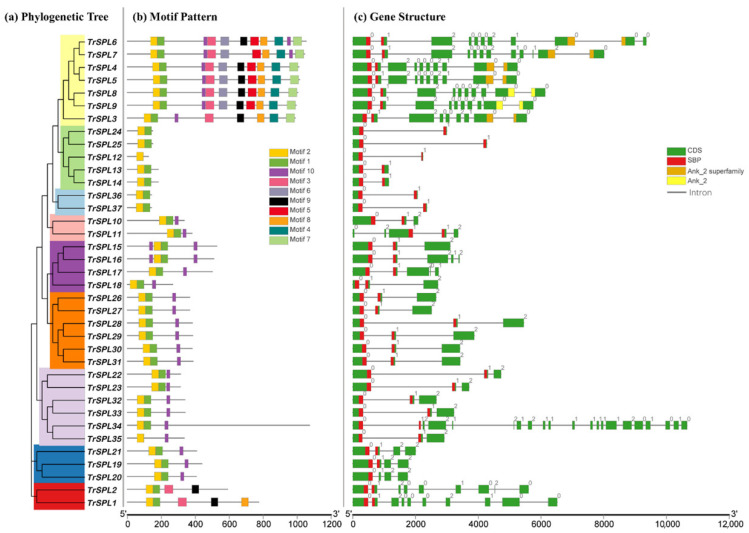
Analysis of conserved motifs and gene structure under the phylogenetic tree of *T. repens SPL* gene family. (**a**) A phylogenetic tree was constructed using the full-length protein sequence of *T. repens SPL* gene family. Different evolutionary clades were marked with different colors. (**b**) Conserved motifs predicted in *TrSPL* proteins. The 10 motifs are represented by squares of different colors. (**c**) Exons and introns are represented by colored squares and black lines. The SBP conserved domain and Ankyrin repeat region were clearly marked and 0, 1, and 2 indicate intron phase.

In order to analyze the diversity and similarity of *TrSPL* gene structure, 10 kinds of motifs were identified in the MEME website (Figure 4). Among them, motif 1 and motif 2 contained a complete SBP domain and the length of motif ranges from 21 to 50. *TrSPL12* and *TrSPL35* only contained one motif, whereas *TrSPL6* contained 11 motifs. The most conserved part was motif 1 in all *TrSPL* genes. Motif1, motif 2, and motif10 were found in 67 percent of *TrSPL* genes. Motifs 4–7 only appeared in *TrSPL3*–*TrSPL9* (in the same clade) which reveal that these motifs were the main factors for the evolution and even functional conservation of this branch. *TrSPL* genes in same branch have similar conserved motifs, indicating they may be having similar function. Sequence information for each motif is provided in Table S4. In the analysis of gene structure, *TrSPL3*–*TrSPL9* have Ankyrin repeat regions (Ank-2 and Ank-2 superfamily), which could be involved in protein-protein interaction [40]. All *TrSPL* genes had at least one intron and *TrSPL34* had the most introns (with 20 introns).

### 3.4. Chromosomal Locations and Synteny Analysis of TrSPL Genes

**Figure 5:**
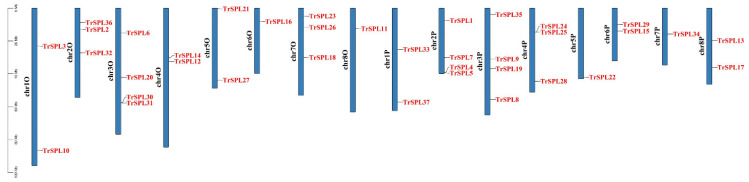
Distribution of *TrSPL* genes on chromosomes. The leftmost scale shows the length of chromosomes. Chromosomes are represented by blue bars.

Thirty-seven *TrSPL* genes were accurately mapped onto *T. repens* chromosomes (Figure 5, Table S5). *TrSPL* genes were relatively evenly distributed on all 16 chromosomes, and the number of *TrSPL* gene on each chromosome ranged from one (Chr6O, chr8O, chr5P, and chr7P) to four (chr3O, chr2P, and chr3P).

**Figure 6:**
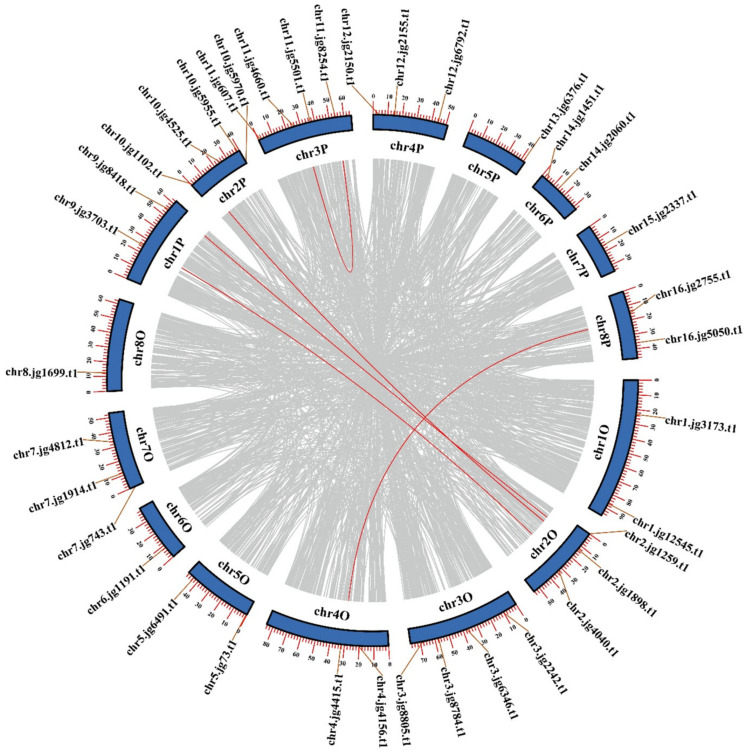
Segmental repeat analysis of *SPL* genes in *T.* *repens* genome. The 16 chromosomes form a circle, and the red lines represent the syntenic region of the 37 *TrSPL* genes. All synteny block produced by doubling the genome are indicated by gray lines.

Gene duplication event is an important way to produce new genes with similar or different functions. We visualized the intraspecific replication events of *TrSPL* genes in Figure 6. A total of five pairs of segmental duplication genes were found, while there was no tandem duplication in the *TrSPL* gene family (Table S6), indicating that segmental duplication plays a very important role in *TrSPL* gene family expansion.

**Figure 7:**
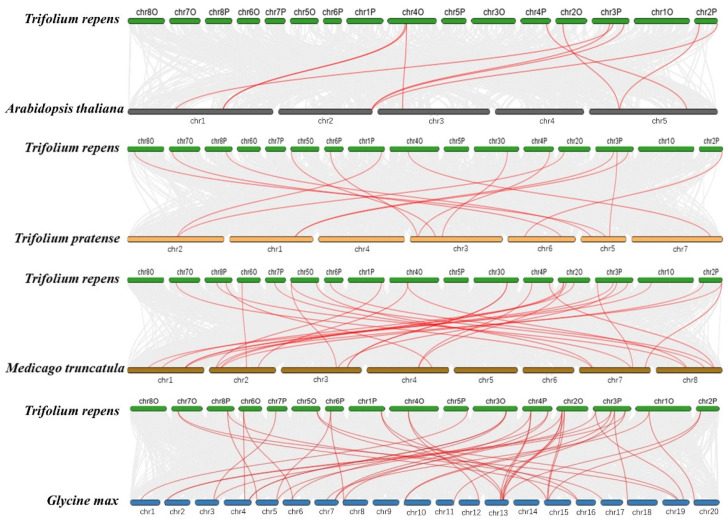
Collinearity analysis of *SPL* gene families between *T.* *repens* and representative species. The red line showed the collinearity of *SPL* gene family in *T.* *repens* and corresponding representative species. Other collinearity between genomes is indicated by gray lines.

To further explore the evolution of the *TrSPL* gene family, four comparative syntenic maps consisted of *A. thaliana*, *Trifolium pratense*, *M. truncatula*, and *Glycine max* were constructed based on collinearity analysis (Figure 7). The number of homologous pairs between *T. repens* and other 4 species was 10 (*A. thaliana*), 14 (Red clover), 28 (*M. truncatula*), and 42 (soybean). The details of homologous pairs are given in Table S7. The comparison results showed that there are more homologous genes between *T. repens* and leguminosae species.

### 3.5. Expression Patterns of TrSPL Genes in Different Inflorescence Development Stage

**Figure 8:**
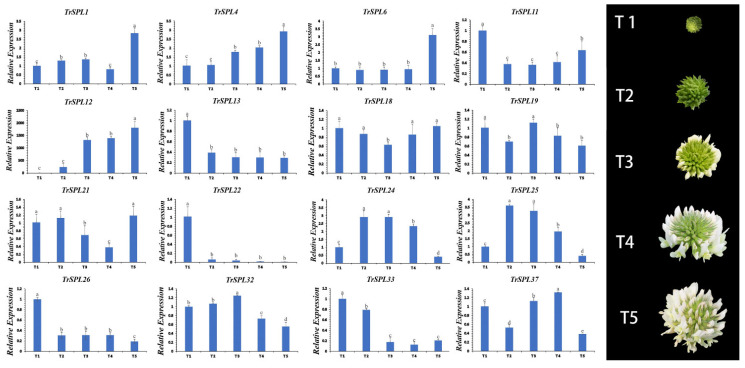
Expression profiles of 16 selected *TrSPL* genes at 5 different flower development stages in *T.* *repens*. T1, Immature inflorescence; T2, Inflorescences in which no floret was open; T3, Inflorescences in which outermost circle of florets were open; T4, Inflorescences in which 50% of florets were open; and T5, Mature inflorescence. The vertical bar is the standard deviation. The letters indicate differences.

In order to further forecast the function of *TrSPL* genes, 16 representative *TrSPL* genes were selected based on phylogenetic tree (Figure 1). By constructing expression profiles in five different inflorescence development stages, preliminarily predicted function of genes was detected (Figure 8). *TrSPL11*, *TrSPL13*, *TrSPL22*, and *TrSPL26* had high expression only in the first development stage (T1) and expression decreased in subsequent stages. *TrSPL33* was highly expressed at T1 and T2, and decreased sharply at three stages after florets bloom. These results suggested that these genes may play an important role in the early development of *T. repens* inflorescence. Of course, some genes, such as *TrSPL1* and *TrSPL6*, were highly expressed only at inflorescence maturity (T5). With the development of inflorescence, the expression level of *TrSPL4* and *TrSPL12* gradually increased and reached the highest at inflorescence maturity (T5). It was worth noting that the relative expression of *TrSPL12* was the highest among the 16 genes, which may be closely related to the regulation of inflorescence development. The expression level of *TrSPL24* and *TrSPL25* increased sharply at T2 stage, and then decreased gradually with the development of inflorescences. The gene expression profile of *TrSPL* genes provided important information to determine the potential regulatory function of *T. repens SPL* gene family in inflorescence development.

## 4. Discussion

*T. repens* is high-quality leguminous forage, and has important economic value in temperate agricultural system [41]. However, flowering directly affects the quality and yield of *T. repens*, and inflorescence development directly affects seed production. The *SPL* gene family is a plant-specific transcription factor family containing a highly conserved SBP domain (76 amino acids), which can bind DNA in a sequence-specific manner and regulate transcription. *SPL* genes can specifically bind related motifs in *SQUAMOSA* promoter of snapdragon and *AP1* promoter of *A. thaliana*, which have been proved to play an important role in regulating plant growth and development [16,42,43]. In this study, 37 *TrSPL* genes were identified in *T. repens*, and much more than 16 in *A. thaliana*, 19 in rice [21], 14 in barley (*Hordeum vulgare*) [38], and 27 in apple (*Malus domestica*) [44], but fewer than 56 in wheat [24], 57 in mustard (*Brassica juncea*) [45], 48 in walnut (*Juglans regia*) [46], 77 in euphorbiaceae [39], and 58 in oilseed rape (*Brassica napus*) [47]. Generally, the number of *SPL* genes are partly affected by the genome size and heterologous polyploidization events. Although the genomes of *A. thaliana* (125 Mb) [48], rice (389 Mb) [49], and apple (632.4 Mb) [50] are much smaller than *T. repens* (1174 Mb) [12], the genome of *B. juncea* (1056.53 Mb) [45,51] and *B. juncea* (1033 Mb) [52] were also smaller than *T. repens*. *T. repens* is an allotetraploid leguminous forage which was predicted that heterologous polyploidization event occurred in the last great glacier period [53]. Interestingly, *B. juncea* and *Brassica napus* are also allotetraploid species. Similar to *T. repens*, they were formed by heterologous polyploidization events through natural hybridization of two diploid ancestral species [51,54]. This may be the main reason why they have a large number of *SPL* genes. Additionally, five pairs of segmental repeat genes were found while no tandem repeat gene pairs, which indicated that segmental repeat is more conducive to the evolution and population expansion of *T. repens SPL* gene family.

The isoelectric point, relative molecular weight and protein sequence length analysis of *TrSPL* genes showed that rich variation within this gene family. A large number of cis-acting elements related to light, hormone and stress response were found, which speculated that the functions of this gene family in *T. repens* are diverse and may play a regulatory role in this physiological process. Furthermore, the *TrSPL* gene showed similar gene structure and conserved motifs in the same clade, but there were significant differences among clades. Ankyrin repeat regions were only found in *TrSPL*3–*TrSPL*9, indicating that these genes in this clade may play an important role in protein-protein interaction [40]. Owing to the ancestor, *SPL* originally formed into two different lineages, named clade I and clade II [55]. Therefore, *TrSPL*1–*TrSPL*9 may belong to clade I due to having more exons and longer protein sequences. Based on the phylogenetic trees of *SPL* gene families of white clover, red clover, *M. truncatula*, and *A. thaliana* further reveal the phylogenetic relationship between them. Moreover, collinearity analysis has been performed between *T. repens* and *T. pratense*, *M. trunculata*, *A. thaliana* or *G. max*. There were only ten pairs of homologous between *T. repens* and *A. thaliana*, while more homologous were found in leguminous species, indicating that the evolution of *SPL* gene in leguminous also had high conservation and homology.

Generally, genes in the same branch of the phylogenetic tree have the similar function. Gene expression patterns can provide crucial information for determining gene function prediction [56]. The expression of *SPL* genes has high specificity in flowering regulation. Most *M. trunculata SPL* genes were highly expressed in flowers, pods, and seeds, but less so in roots, stems, and leaves [26]. In wheat, most *TaSPL* genes were found to regulate the development of inflorescence and spike. Some genes (*TaSPL034*, *TaSPL035*, *TaSPL037 TaSPL044*, and *TaSPL052*) are only expressed in inflorescences, but almost not expressed in roots, stems, and leaves [24]. More than half of *OsSPL* genes were expressed in young panicles of rice [21]. In *Prunus mume*, most of the *SPL* genes showed high transcript levels in flower buds and young fruit. The expression of *PmSBP7* in pistil is 12 times higher than that in other tissues [57]. In this study, Previous studies have shown that the *A. thaliana SPL* gene in clade V (*AtSPL3*) and clade VI (*AtSPL2*, *AtSPL10*, and *AtSPL11*) could regulate flowering time [18,58], and it was speculated that *TrSPL19-25* located in the same clade may have similar functions in regulating the flowering time of *T. repens*. Interestingly, the light response elements were detected in all of these genes. Similarly, the *A. thaliana SPL* genes (*AtSPL8*, *AtSPL9*, and *AtSPL15*) in clade III and clade VIII have been proved to affect inflorescences development [19,20], and *TrSPL10* to *14* and *TrSPL32* to *35* (assigned into clade III and clade VIII) were possible relevance to inflorescences development of *T. repens*. Among these genes, *TrSPL11* and *TrSPL13* was highly expressed only at T1 stage, and *TrSPL33* was highly expressed at T1 and T2 stages, indicating that these genes play an important regulatory role in the early development of *T. repens* inflorescences. Specially, with the development of *T. repens* inflorescences, the expression of *TrSPL12* gradually increased and peaked at T5, indicating that *TrSPL12* may play an important effect with the development of inflorescences. *TrSPL22* was highly expressed only at the beginning of inflorescence development, but almost not at other stages. In brief, *TrSPL* gene family is such important in *T. repens* flowering regulation, especially in inflorescence development.

In this study, except for regulating flowering time and inflorescence development, the discovery of hormones and external stress responsive elements also indicates the diversity of *TrSPL* gene family functions. *AtSPL6* can bind to the nuclear localization immune receptor to activate the defense transcriptome and generate defense signals against pathogens [59]. *AtSPL* genes (*AtSPL1*, *AtSPL12*, and *AtSPL14*) have been proved to be involved in regulating the development and its sensitivity to fumonisin B1 of *A. thaliana*. Similarly, *Vpsbp5* in the same clade has also been proved to prevent powdery mildew in grapes [60]. In the same branch, methyl jasmonate response elements and salicylic acid response elements were found upstream of some TrSPL genes. Salicylic acid and methyl jasmonate are important immune hormones in plants [61]. The results showed that *TrSPL* genes in cade II and IV may play an important role in enhancing pathogen defense response in *T. repens*. Previous reports have indicated that *SPL* gene family is also involved in abiotic stress response [62,63,64,65]. Both low-temperature response elements and stress response elements were found upstream of *TrSPL21*, *TrSPL25*, and *TrSPL28*.

## 5. Conclusions

*T. repens* is the most widely cultivated perennial legume forage in temperate regions around the world. However, flowering time and inflorescence development directly affects the quality and yield, as well as seed production. The *SPL* gene family is a plant specific transcription factor family, which has been proved to play a critical role in regulating plant formation time and development of flowers. In this study, a total of 37 *TrSPL* genes were identified from the whole genome of *T. repens* and were divided into nine clades based on phylogenetic tree. Seventeen *TrSPL* genes have potential target sites for miR156. The basic information of 37 *TrSPL* genes was obtained, including isoelectric point (PI), relative molecular weight (MW), protein sequence length (aa), and subcellular localization. The result of cis acting element analysis showed that a large number of action elements in response to light were identified and potential flowering regulation function was predicted. Moreover, 37 *TrSPL* genes were relatively dispersedly distributed on 16 chromosomes, and 5 pairs of segmental repeat genes were found, which indicated that segmental duplication was the main way of *TrSPL* gene expansion. Furthermore, the gene expression profiling showed that *TrSPL11*, *TrSPL13*, TrSPL2,2 and *TrSPL26* were highly expressed only in the early stage of inflorescence development, while *TrSPL1* and *TrSPL6* are highly expressed only in the mature inflorescence. The results of this study will provide valuable clues for candidate gene selection and elucidating the molecular mechanism of *T. repens* flowering regulation.

## Figures and Tables

**Table 1:** The basic information and subcellular localization of all identified *SPL* genes in *T. repens*.

Name	PI	MW (Da)	Length (aa)	Subcellular Localization
*TrSPL*1	6.12	86,752.27	773	nucleus
*TrSPL*2	6.88	67,153.17	591	nucleus
*TrSPL*3	8.18	109,672.44	989	plasma membrane
*TrSPL*4	5.75	111,233.87	1010	endomembrane system
*TrSPL*5	5.72	111,303.94	1014	endomembrane system
*TrSPL*6	7.01	116,204.16	1053	nucleus
*TrSPL*7	8.51	115,211.08	1044	endomembrane system
*TrSPL*8	5.8	111,853.78	1004	nucleus
*TrSPL*9	5.86	110,745.6	995	nucleus
*TrSPL*10	8.92	37,297.64	335	nucleus
*TrSPL*11	9.14	40,922.71	363	nucleus
*TrSPL*12	6.9	14,269.05	124	nucleus
*TrSPL*13	9.3	20,662.94	182	nucleus
*TrSPL*14	9.41	20,574.88	182	nucleus
*TrSPL*15	5.98	57,844.27	527	nucleus
*TrSPL*16	6.46	55,524.04	509	nucleus
*TrSPL*17	6.31	55,948.26	500	nucleus
*TrSPL*18	8.97	30,286.98	267	chloroplast
*TrSPL*19	8.88	48,472.58	439	nucleus
*TrSPL*20	8.73	44,554.3	406	nucleus
*TrSPL*21	8.17	45,859.11	408	nucleus
*TrSPL*22	8.67	34,247.9	313	nucleus
*TrSPL*23	8.67	34,313.96	312	nucleus
*TrSPL*24	6.1	17,499.07	149	nucleus
*TrSPL*25	6.1	17,472.04	149	nucleus
*TrSPL*26	7.6	41,040.79	367	nucleus
*TrSPL*27	7.61	40,962.63	367	nucleus
*TrSPL*28	8.86	42,684.84	383	nucleus
*TrSPL*29	8.62	42,817.9	385	nucleus
*TrSPL*30	6.62	42,612.13	382	nucleus
*TrSPL*31	6.78	42,977.52	387	nucleus
*TrSPL*32	9.2	37,707.11	339	chloroplast
*TrSPL*33	9.41	37,953.47	340	nucleus
*TrSPL*34	6.99	37,614.66	343	plasma membrane
*TrSPL*35	8.73	36,551.32	335	nucleus
*TrSPL*36	7.04	16,415.99	142	nucleus
*TrSPL*37	7.04	16,430.02	142	nucleus

## Data Availability

All data generated or analyzed during this study are included in this article and its attached documents. The *T. repens* genome resources were downloaded from the EMBL/GenBank data libraries (Bioproject PRJNA523044, https://www.ncbi.nlm.nih.gov/genbank/, accessed on 5 March 2021) (Griffiths et al., 2019). The genome data used for comparative syntenic analysis were obtained from open database. The genome data of *A. thaliana* were downloaded from TAIR (https://www.arabidopsis.org/download/index-auto.jsp?dir=%2Fdownload_files%2FSequences%2FAraport11_blastsets, accessed on 10 June 2021). The genome data of *M. truncatula* were downloaded from the *M. truncatula* Genome Database (http://www.medicagogenome.org/, accessed on 10 June 2021). The genome data of *Trifolium pratense* were downloaded from Ensembl website (https://plants.ensembl.org/index.html, accessed on 10 June 2021). The genome data of *G. max* were downloaded from the *G. max* Genome Database (https://www.soybase.org/, accessed on 15 June 2021).

## Supplementary Materials

The following supporting information can be downloaded at https://www.mdpi.com/article/10.3390/genes13050900/s1. Table S1: The name and sequence information of primers involved in this study. Table S2: Cis acting element analysis of 37 *SPL* genes in white clover. Table S3: The miR156 target information of *TrSPL* genes. Table S4: Analysis of conserved motifs of SPL protein in White clover. Table S5: Specific location information of *TrSPL* genes on chromosome. Table S6: Segmental duplication analysis of *SPL* genes in white clover genome. Table S7: Orthologous relationships table of white clover and *A. thaliana*. Figure S1: Analysis of 2000 bp cis acting element upstream of *TrSPL* genes. Different action elements are represented by blocks of different colors.

## Abbreviations

SPL	Squa promoter binding protein–like
SBP	Squamosa promoter binding protein
CDS	Coding sequence
HMM	Hidden Markov model
MW	Molecular weight
PI	Isoelectric point
NLS	Nuclear localization signal
