# Supplementary figures and images for: Genome-Wide Identification, Characterization, and Expression Profiling Analysis of SPL Gene Family during the Inflorescence Development in Trifolium repens
Source: Genes (Basel). 2022 May 18;13(5):900. doi: 10.3390/genes13050900 (PMC9140761; doi:10.3390/genes13050900)

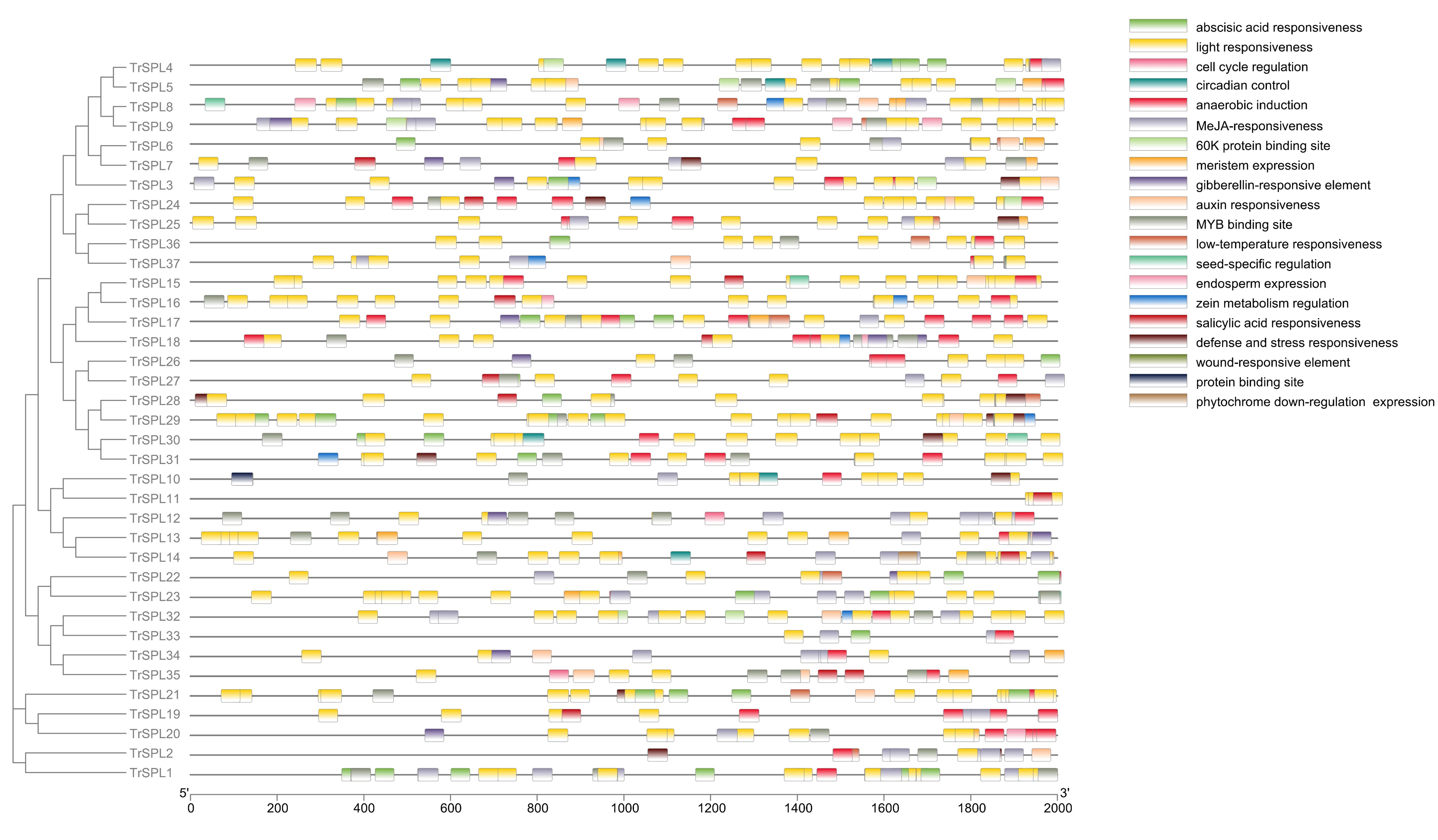

Supplement: Supplementary file 1 [file genes-13-00900-s001.zip › Figure S1.tif]
